# Preanalytical Errors in Clinical Laboratory Testing at a Glance: Source and Control Measures

**DOI:** 10.7759/cureus.57243

**Published:** 2024-03-30

**Authors:** Nani Nordin, Siti Nadirah Ab Rahim, Wan Farhana Azwanee Wan Omar, Sarah Zulkarnain, Susmita Sinha, Santosh Kumar, Mainul Haque

**Affiliations:** 1 Pathology, Faculty of Medicine and Defence Health, National Defence University of Malaysia, Kuala Lumpur, MYS; 2 Physiology, Khulna City Medical College and Hospital, Khulna, BGD; 3 Periodontology and Implantology, Karnavati School of Dentistry, Karnavati University, Gandhinagar, IND; 4 Karnavati Scientific Research Center (KSRC), Karnavati School of Dentistry, Karnavati University, Gandhinagar, IND; 5 Pharmacology and Therapeutics, National Defence University of Malaysia, Kuala Lumpur, MYS

**Keywords:** biological variation, laboratory quality, blood sample quality, laboratory sample rejection, phelebotomy, hemolysis, laboratory process, laboratory error, pre-analytical variables, results inaccuracy

## Abstract

The accuracy of diagnostic results in clinical laboratory testing is paramount for informed healthcare decisions and effective patient care. While the focus has traditionally been on the analytical phase, attention has shifted towards optimizing the preanalytical phase due to its significant contribution to total laboratory errors. This review highlights preanalytical errors, their sources, and control measures to improve the quality of laboratory testing. Blood sample quality is a critical concern, with factors such as hemolysis, lipemia, and icterus leading to erroneous results. Sources of preanalytical errors encompass inappropriate test requests, patient preparation lapses, and errors during sample collection, handling, and transportation. Mitigating these errors includes harmonization efforts, education and training programs, automated methods for sample quality assessment, and quality monitoring. Collaboration between laboratory personnel and healthcare professionals is crucial for implementing and sustaining these measures to enhance the accuracy and reliability of diagnostic results, ultimately improving patient care.

## Introduction and background

The accuracy of diagnostic results is of prime importance as it serves as the backbone for healthcare decisions and patient care. It is commonly perceived that the performance of a diagnostic laboratory is reflected by quality during the analytical phase. However, over the years, the focus of laboratory performance has expanded from ensuring the precision of the analytical phase to optimization of the preanalytical phase.

The laboratory total testing process (TTP) comprises preanalytical, analytical, and postanalytical phases. The preanalytical phase occurs from as early as test ordering (pre-pre analytical phase) to the point where the sample is ready for analysis. Preanalytical errors contribute to around 60%-70% of laboratory errors [1,2]. This is owing to the involvement of activities that take place outside the laboratory and the involvement of manual handling of the specimen during this phase. Preanalytical errors can significantly affect the reliability and accuracy of the test results, compromising patient care and increasing the financial burden of healthcare.

Problem statement

Despite increasing awareness among laboratorians, preanalytical errors remain a significant concern in laboratory practice, contributing to a substantial portion of total laboratory errors. Addressing these issues is essential to ensure the quality of laboratory testing and improve patient care outcomes.

The objective of the study

The objective of this study is to comprehensively review factors contributing to preanalytical errors in clinical laboratory testing and explore current measures to alleviate these errors. This review aims to raise awareness among healthcare professionals and laboratory personnel of the importance of preanalytical errors and their collaborative roles in implementing good laboratory practices and ultimately enhancing the accuracy and reliability of diagnostic results.

## Review

Materials and methods

**Figure 1:**
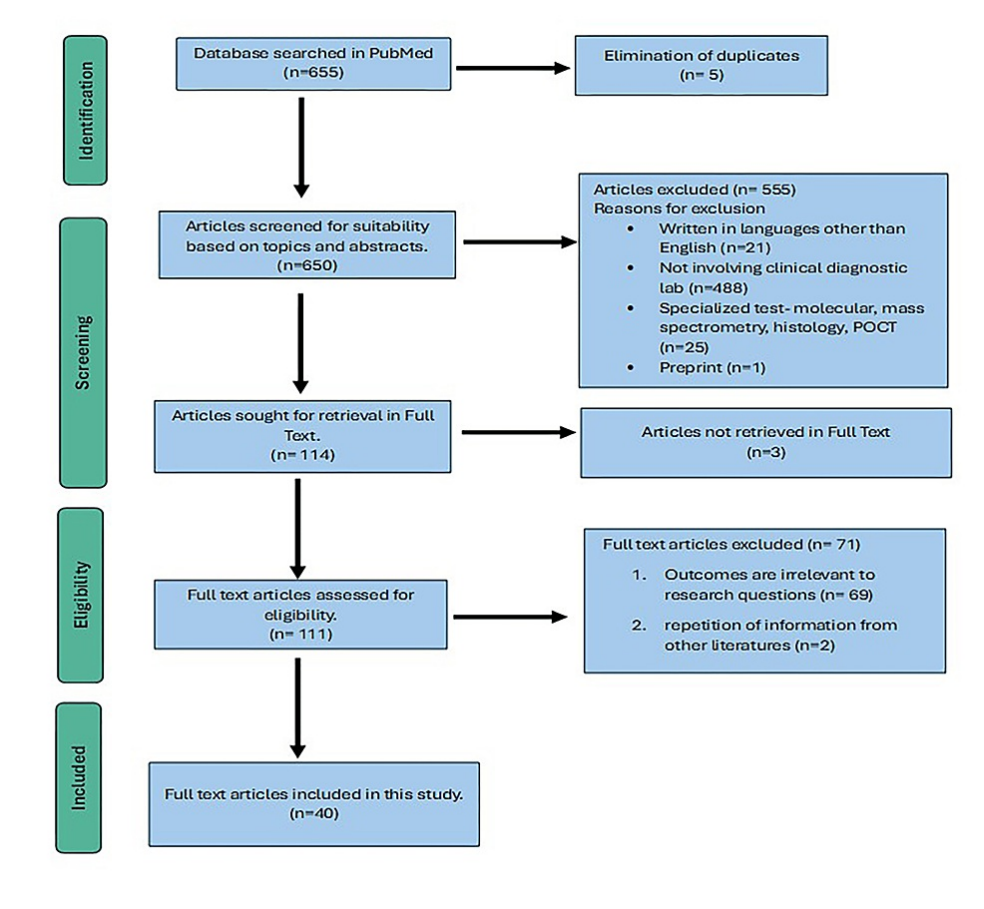
Flow chart illustration of steps for article selections using the Preferred Reporting Items for Systematic Reviews and Meta-Analyses (PRISMA) 2020 guideline. Notes: Prisma Guidelines [3]. POCT=Point-of-Care Testing Image Credit: Nani Nordin

This paper's primary literature review source was the PubMed online database. The keywords "laboratory error," "preanalytical error," and "preanalytical variables" were utilized in the literature search. The search was set to publication date over the last 10 years. However, most data were gathered from the literature published over the previous five years to ensure the most up-to-date information. Various types of literature, including original articles, systematic reviews, and narrative reviews, were scrutinized. Forty articles most applicable to the objective of this review were selected. The following flow chart depicts the steps of the literature search in this review article according to the Preferred Reporting Items for Systematic Reviews and Meta-Analyses (PRISMA) 2020 flow diagram (Figure 1) [3]. A total of 655 articles were yielded from the PubMed online search. Six-hundred and fifty articles were further screened after the elimination of duplicates. Screening was done based on topics and abstracts, and availability in full text resulted in the retrieval of 111 eligible articles. Forty articles relevant to the research objective were included after further evaluation. The following flow chart depicts the steps of the literature search described above.

Blood sample quality

Poor blood sample quality is the essence of the preanalytical variable, contributing to 80%-90% of preanalytical errors [4]. Sample quality can be in the form of in vitro biological changes in the blood composition (e.g., due to inappropriate patient preparation before blood sample collection) or in vivo changes in blood composition (e.g., improper sample collection and handling). Most literature concluded that hemolyzed samples are the primary source of poor blood sample quality (40-70%), followed by inappropriate sample volume (10-20%), the use of the wrong container (5-15%), and clotted sample (5-10%) [4].

Hemolysis, lipemic, and icteric sample

From a preanalytical error perspective, the hemolyzed sample mainly refers to the in-vivo breakdown of red blood cells (RBC) reflected by increased concentrations of cell-free hemoglobin in serum or plasma [5]. It occurs due to non-biological conditions, particularly during sample collection and handling. Hemolysis causes spurious release of intracellular analytes such as potassium, magnesium, phosphate, enzymes such as lactate dehydrogenase (LDH), aspartate and alanine transaminases (ALT, AST), and dilution of extracellular components such as Na^+^. Furthermore, changes in spectral absorbance due to the presence of cell-free hemoglobin interfere with various biochemistry analytes measured using the spectrophotometry method [5]. Other forms of deterioration of sample quality are icteric and lipemic changes. Bilirubin in icteric samples interferes with peroxidase-coupled reactions, causing a falsely low glucose, cholesterol, triglyceride, and uric acid measurement [6]. Lipemia is defined as turbidity in a sample caused by the accumulation of lipoproteins, mainly very-low-density lipoproteins (VLDL) and chylomicrons. It causes an increase in the solid phase relative to the water phase component in the patient's serum samples, leading to pseudo hyponatremia, which occurs mainly in the indirect ion-selective electrodes (ISE) measurement method [7]. Additionally, some biochemistry analytes such as creatinine, potassium, sodium, chloride glucose, direct bilirubin, and urea will give variable results due to spectral interference and volume displacement effect of lipemia [6,8].

Factors contribute to preanalytical errors

**Figure 2:**
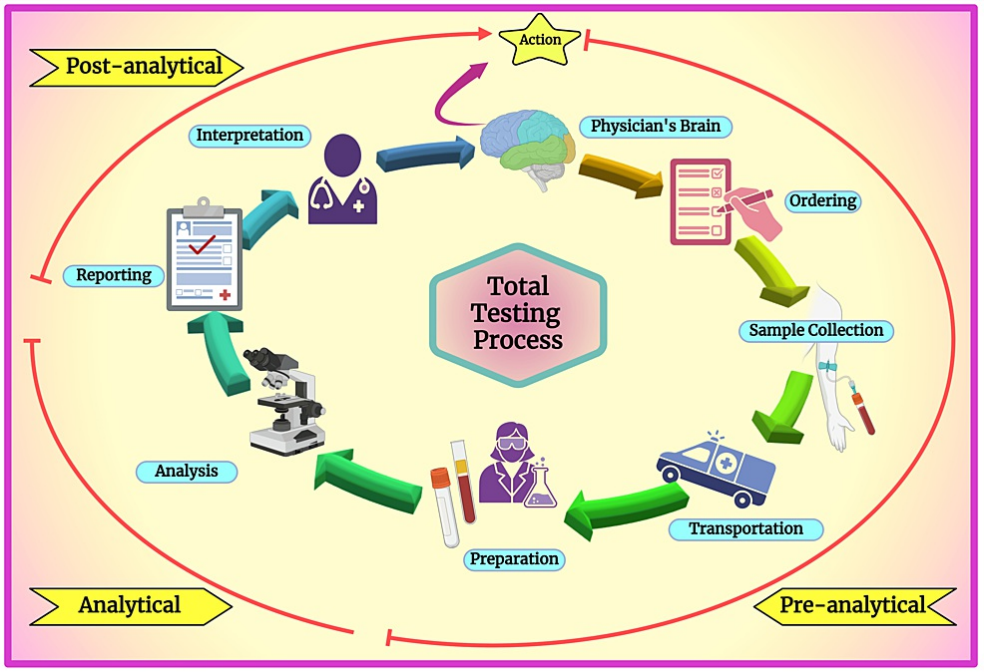
Schematic diagram showing the total testing process. Notes: This figure was drawn using the premium version of BioRender [9] (license number WG26KTF43S). Image Credit: Susmita Sinha

A complete laboratory process has been described as a brain-to-brain loop concept [2]. This concept signifies the physician's brain, which is involved in the first step of the laboratory process, where the selection of tests is made, and in the final step, where the result is transmitted (Figure 2).

The preanalytical, analytical, and postanalytical phases are spread between the first and final steps. This concept led to a more systematic error identification and classification associated with laboratory performance. Table 1 describes the source of errors classified based on the phase of the testing process.

**Table 1:** Source of laboratory errors and their distributions within the different phases of the testing processes.

The phase of the testing process	Source of error
Pre-analytical [1,2,10,11]	a. Inappropriate test request; b.Order entry errors; c. Misplaced test request form; d. Unable to determine test requester; e. Patient misidentification; f. Inappropriate tube Improper sample collection (hemolysis, clotting, insufficient volume); g. Sample collected from the infusion site; h. Improper sample handling, storage, and transportation; i. Sample labeling error; j. Sorting and routing errors; k.Sample processing errors (centrifugation, decapping, aliquoting, etc.)
Analytical [2,11]	a. Sample loss; b. Sample mix-up/ interference; c. Undetected failure in quality control; d. Equipment malfunction analytical errors
Post-analytical [11]	a. Test result loss; b. Erroneous validation of test results; c. Transcription error; d. Incorrect result interpretation

It is acknowledged that most sources of laboratory errors are from the preanalytical phase. The essential sources of errors in the preanalytical phase are as follows:

(a) Inappropriate test request (pre-preanalytical)

Procedures involving test requests are categorized as the pre-preanalytical phase. Inappropriate laboratory tests may be ordered by unnecessary tests (overuse) or by not ordering needed tests (underuse). Inappropriate test requests impact total costs and increase the risk of medical errors [10,12]. The estimations of inappropriate laboratory tests vary within 11-70% for general biochemistry and hematology tests, 5-95% for urine screens and microbiology, and 17.4-55% for cardiac enzymes and thyroid tests [13]. The lack of involvement of laboratory specialists in selecting a test is believed to be one of the reasons.

(b) Patient's preparation

Fasting is one of the most critical patient preparations required before sample collection. It is necessary for some tests, such as glucose and cholesterol tests. Marked metabolic and hormonal changes occur in response to food ingestion, principally due to the absorption of fluid, macronutrients, and other food constituents [14]. The duration of the fasting requirement varies from 8 to 12 hours, depending on the test. Failure to adequately fast results in falsely high values of various analytes, mainly glucose and triglyceride [1,14]. Lipemic samples may occur if the blood sample is collected after a heavy meal. This may cause a false high cholesterol result and interfere with the optical measurement method used in various electrolyte analyses [1].

Cigarette smoking and alcohol consumption results in a marked increase in triglyceride-rich lipoprotein metabolic rate. Coffee has been shown to increase glucose concentration. All these need to be avoided before blood collection [14]. Chewing gum has been demonstrated to affect various tests due to its ingredients, such as glycerol and butylated hydroxy anisole (BHA), and the stimulation of gastric secretion during chewing [15]. Various guidelines recommended the prohibition of chewing gum before blood collection [11].

Certain drugs can interfere with blood results. Literature reports the prevalence of drug-laboratory test interactions (DLTI) of up to 43% [16]. The increasing usage of over-the-counter (OTC) drugs, herbal preparations, and dietary supplements contributes to the prevalence. Drugs can affect blood results by physiological interference through unwanted effects or analytical interference, which is method-dependent [16]. Recently, the interference of biotin on immunoassays that utilized the streptavidin-biotin system has gained popularity. Besides therapeutic use, biotin is found mainly in various dietary supplements for hair and nail health [17]. Upon blood investigation, it is essential to be informed of any drugs the patient consumes.

(c) Sample collection

The sample collection process is critical as it could contribute to the bulk of preanalytical errors. This involves specimen labeling and patient identification, collection techniques, and transportation before analysis [10].

Patient identification and Tube Labelling

It has been determined that 16% of errors in the phlebotomy process happen due to patient misidentification, and 56% are due to improper labeling [18]. Electronic specimen labeling with automated links to patients is among the measures to alleviate this risk [18]. The labeling process must be performed in the patient's presence. Guidelines have suggested that patient identification procedures be done using open-ended questions to the patient and mandate a minimum of two identifiers that include the patient's full name [18,19].

Patient's Position

Changing position from lying to standing and vice versa has been shown to affect various analytes. This is due to the gravitational effect of the blood at different positions. The patient should remain in the same position for at least 15 minutes before blood collection [20].

Diurnal Variation

Timing of blood collection is essential due to diurnal variation exhibited by various analytes such as cortisol and adrenocorticotropin. Serum iron levels may change by 30%-50% within a day [21]. It is recommended that blood samples are collected in the morning as many analytes with diurnal variation have their reference values set at 7-9 AM [1].

Phlebotomy Techniques

Proper techniques during blood collection are essential to prevent hemolysis, sample contamination, or other preanalytical errors that could compromise the quality of the specimen. Pseudohyperkalemia is among the frequently observed abnormalities following non-adherence to proper techniques. These include excessive fist clenching, prolonged tourniquet, use of too small needle size, forceful blood extraction, and vigorous sample mixing, leading to local tissue and cellular damage resulting in hyperkalemia [22]. Their impact on potassium is even more significant than sample contamination from the wrong draw order [23]. Venous stasis following prolonged tourniquet over three minutes is known to cause up to 12.4% elevation of analytes such as total protein, albumin, and protein-bound analytes such as total Ca^2+^ [24]. A transillumination device to better visualize the vein has been suggested to reduce errors associated with prolonged tourniquets [25].

Order of Draw

Blood samples must be drawn in a specific order when multiple types of tubes are used. The Seventh Edition of the Clinical and Laboratory Standard Institute (CLSI) suggested that the order of draw sequence should start with blood culture or sterile tubes, followed by plain or gel tubes and then tubes containing additives, namely, the heparin tubes, ethylenediaminetetraacetic acid (EDTA) tubes, and the glycolytic inhibitor or fluoride tubes [26]. The purpose is to reduce the carryover of additive contamination, which can lead to erroneous results [26,27]. EDTA contamination, for example, can cause spurious hyperkalemia and hypocalcemia secondary to the EDTA chelation effect [22,28]. In practice, tubes and additives are identified mainly by the color of the tube closures. Different manufacturers' non-standardized colors of tube closures have created confusion for phlebotomists, which may lead to the wrong draw order.

(d) Sample handling, transportation, and storage

The stability of an analyte in a sample is defined as the preservation of its physiochemical properties over time. The instability of analytes, an invisible aspect of sample quality, is affected by sample handling, including time and temperature during transportation and storage. A whole blood specimen requires centrifugation within two hours after venipuncture to separate serum or plasma from RBC [1]. Before that, complete blood clots must be ensured, and the timing depends on the types of tubes used. After separation, the sample will generally be stable at room temperature for eight hours and up to 48 hours at 2-4 °C. After 48 hours, the serum specimen should be frozen at -20 °C [1]. It is mandatory for the laboratory to have a procedure for time and temperature monitoring and to specify a time limit from sample collection to analysis [29].

Measures to improve preanalytical errors

**Figure 3:**
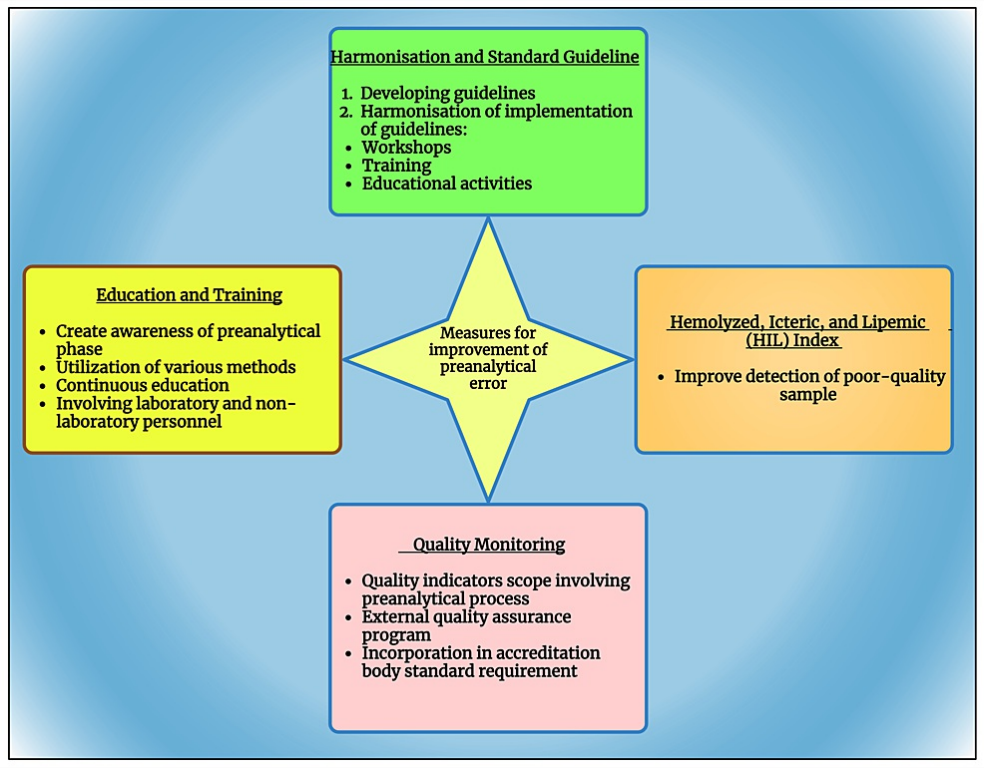
Schematic diagram showing the measures to improve preanalytical error. Notes: This figure was been drawn with the premium version of BioRender, with the license number LG26KR5WFE. *Harmonization [10]; *Education and training [30,31]; *Hemolytic, icteric, lipemic [4,5,32]; *Quality monitoring [33-35]; *Automation [34,36,37] Image Credit: Susmita Sinha

The initiatives to improve preanalytical took place decades ago after it was first described in 1977. Over the years, more sources of preanalytical error have been studied, and various measures (Figure 3) have been taken to minimize the error.

Harmonization and standardized guideline

Upon awareness of the lack of standardization in many processes in the preanalytical phase, efforts at various levels have been initiated to close this gap. One of the large-scale initiatives is the European Federation of Clinical Chemistry (EFLM) "Preanalytical Phase" working group (WG-PRE), established in 2013. This working group aims to improve harmonization in the preanalytical phase across European and other member societies [10]. Their activities include developing guidelines and recommendations on various aspects of the preanalytical phase and conducting workshops, training, and educational activities [13,20,38-41]. The initiatives provide the framework for standardization and harmonization for laboratories worldwide.

Education and training

It has been observed that the most common reason for errors in the preanalytical setting is human error (82.6%) compared to technical errors at only 4.3% [30,31]. Therefore, education to create awareness of preanalytical errors and training on phlebotomy and specimen handling skills, encompassing all personnel involved in laboratory investigations, are crucial. Despite the availability of guidelines, ensuring implementation and sustaining guideline practices needs attention. Studies have shown that training for medical staff has significantly reduced the percentage of preanalytical errors [30,31]. While training the laboratory personnel is straightforward, training the non-laboratory healthcare personnel on handling blood samples remains challenging. Various training methods, including direct education, e-learning, or providing reports on sample quality, can be used. One study demonstrated that a role-play model for training interns on the importance of the preanalytical phase has shown positive outcomes as it simulates actual situations [1,34]. It is essential that training is continuous to ensure the continuity of transfer of skills and knowledge. Besides knowledge enhancement and training in technical skills, practical communication skills with patients are essential. One study reported a lack of communication with patients in preparation for phlebotomy [31].

Hemolyzed, icteric, and lipemic (HIL) index

Detection of poor sample quality can prevent erroneous results. Traditionally, sample quality evaluation is done by visually inspecting the sample's appearance to ensure its suitability before analysis. Poor sample quality, such as hemolyzed, clotted, insufficient or overfilled samples, and wrong containers, is considered a sample rejection criterion for most laboratories. However, some deterioration in sample quality is invisible to the naked eye and is suspected only when results are spurious. Detection of HIL samples using automated HIL-indices analysis is widely used to allow rating of the degree of HIL, hence better management of the sample.

The hemolytic index (H-index) estimates the potential concentration of cell-free hemoglobin by photometric assessment of serum or plasma at a specific wavelength. Working Group for Preanalytical Phase (WG-PRE) recently released a standardized recommendation for using the H-index, which proposes including comments when releasing results in the hemolyzed sample [4,5,32].

Records of the HIL index assist in communication with the requesting clinician [8]. The clinician should note the detection of an icterus sample earlier as a caution for potential result inaccuracy [6]. Unlike hemolyzed and icteric samples, lipemic samples can be further processed using high-speed or ultracentrifugation to obtain clear serum for analysis. Furthermore, an alternative method, such as direct ISE, can prevent pseudo hyponatremia in lipemic samples [7].

Quality monitoring

Realizing the importance of preanalytical error, many laboratories incorporate the performance of the preanalytical phase as their quality indicator (QI). Studies have proven that this exercise has significantly reduced laboratory error [34,35]. There have been efforts by the International Federation of Clinical Chemistry (IFCC) working group on laboratory errors and patient safety (WG-LEPS) to standardize the implementation of preanalytical error monitoring by developing a model of QI (MQI) [32]. These QI include hemolyzed, clotted, insufficient, and misidentified samples.

Over the past years, most external quality assurance (EQA) providers have incorporated preanalytical schemes to provide a platform for preanalytical quality control monitoring. In their latest update, international accreditation bodies, mainly ISO 15189, have included preanalytical phase monitoring requirements in their standard [38].

Studies have indicated that many laboratories are now collecting data related to preanalytical performance, indicating their awareness of preanalytical errors. However, many did not analyze their data or take further action on their findings, which defeats the purpose of quality monitoring [2,42].

Automation

It is acknowledged that the propensity to error in the preanalytical phase is due to its relatively high involvement of manual procedures [36]. A risk assessment study by Bellini et al. reported that manual processes, such as acceptance of test orders, patients' identification, tube labeling, and sample collection scored the highest risk index [37].

Replacing manual activities with automation has significantly reduced laboratory error, particularly in the analytical and post-analytical phases [34]. The development of Laboratory Information Software (LIS) played a significant role in laboratory automation and has replaced some of the manual processing in the preanalytical phase. Automation in the test ordering process can be obtained by a computerized order entry system, which can directly transmit order information into the LIS, where barcoded labels can be generated and printed for bedside labeling. This can avoid transcription errors, unclear test requests, and errors in patient identification. In addition, interfacing LIS with clinical data allows the filtering of duplicate or inappropriate requests [37].

With the advancement of technology, devices such as automated phlebotomy trays and vein viewers are available to improve phlebotomy. During specimen handling, automated temperature data loggers and radio-frequency chips can help track and monitor temperature effectively during transport. Some robotics systems can automatically load samples on the transport track for delivery to the laboratory, reducing turn-around time and cost [37].

Although some devices are readily available and accepted for use, advancements in information technology are still not widely available. Adopting digital and more integrated healthcare requires workforce readiness and cultural transformation [37]. Automation is often perceived as a threat to human work, creating resistance by some personnel. Education and awareness are mandatory to overcome this. Table 2 illustrates the key findings of this narrative review paper**.**

**Table 2:** Depicting the principal findings of the current paper.

Serial Number	Key Findings
1.	Poor sample integrity is the primary source of preanalytical error.
2.	Human error accounts for the majority of preanalytical errors.
3.	The development of standardized guidelines provides a platform to facilitate good laboratory practice.
4.	Continuous education and training are critical factors in mitigating preanalytical error.
5.	Regulating the participation of various parties involved in the preanalytical phase remains a significant challenge.

Limitations of this study

This review focuses only on the critical sources of preanalytical error. This article may not address various other factors and mitigation measures comprehensively. Furthermore, given the evolving technology in healthcare practice, this article may not have captured the latest emerging technologies in preanalytical error management. Future research exploring emerging trends and challenges with a more robust discussion of technological advancement in this field is the way forward.

## Conclusions

The preanalytical phase of clinical laboratory testing plays a crucial role in ensuring the accuracy and reliability of diagnostic results. Measures to improve this phase should be a collaborative approach between laboratory personnel and healthcare professionals. Ongoing efforts are to standardize preanalytical processes, reduce errors, and enhance the quality of sample collection, handling, and transportation. Healthcare professionals, laboratory staff, and researchers should remain vigilant and up-to-date on the latest findings in this field to continually improve the quality of patient care through precise laboratory testing.
